# Fabrication of 3D GelMA Scaffolds Using Agarose Microgel Embedded Printing

**DOI:** 10.3390/mi13030469

**Published:** 2022-03-18

**Authors:** Bo Yang, Tianqi Liu, Ge Gao, Xianglin Zhang, Bin Wu

**Affiliations:** 1 State Key Laboratory of Materials Processing and Die & Mould Technology, School of Materials Science and Engineering, Huazhong University of Science and Technology, Wuhan 430074, China; m202071032@hust.edu.cn (B.Y.); ltqfxclhl@foxmail.com (T.L.); hust_zxl@mail.hust.edu.cn (X.Z.); 2 School of Medical Technology, Beijing Institute of Technology, Beijing 100081, China; gaoge@bit.edu.cn

**Keywords:** tissue engineering, embedded bioprinting, GelMA, agarose microgel, printability, scaffold

## Abstract

Photocrosslinked Gelatin–Methacryloyl (GelMA) has been widely used in the field of 3D bioprinting due to its excellent biological properties, but its properties are not yet optimized. With the advent of embedded printing, the balance between hydrogel printability and cell viability is expected to be achieved. Agarose microgel is a good support material because of its simple preparation, good biocompatibility, high melting point, and good rheology. In this study, aiming at realizing a GelMA/Agarose suspension printing system, the printing effect of the suspension process was explored, and a suitable process printing window was defined. The resulting scaffolds showed better water absorption and elasticity, but larger deformation during printing. This study explored some potential roles of suspension baths in embedded printing, paving the way for the preparation of good suspension structures that can be convenient for customized tissue engineering applications.

## 1. Introduction

Hydrogel scaffolds have been widely used in tissue engineering and 3D cell culture because hydrogels, as mimetics of the extracellular matrix [1], can provide an aqueous environment and facilitate the transport of nutrients, cell adhesion, growth, and differentiation. Traditional scaffold materials (metals, ceramics, polymers) do not have these advantages [2]. With the application of the 3D printing technology in the field of tissue engineering, the preparation of hydrogel scaffolds has also entered the era of customization, becoming applicable in various fields such as personalized tissue repair of blood vessels [3], valves, skin [4], etc. Commonly used hydrogel materials include alginate, collagen, gelatin, chitosan. For example, Shi Lei [5] and others used sodium alginate and gelatin to prepare multi-step cross-linked skin tissue scaffolds. Nocera [6] et al. prepared porous collagen scaffolds under neutral pH conditions and demonstrated their ability to promote cell growth and differentiation. However, sodium alginate lacks cell binding sites and has poor biocompatibility. The printing ability and structural properties of collagen are limited. In order to obtain better tissue engineering scaffolds, ideal hydrogels need to have both good cell compatibility and 3D printing suitability. Gelatin–Methacryloyl (GelMA) is a photocrosslinked hydrogel [7] with good biocompatibility, degradability [8], and mechanical properties after crosslinking and has become one of the most widely used materials in biomanufacturing.

However, GelMA has also some drawbacks. The temperature-sensitive properties of GelMA make it difficult to obtain stable printability. When the temperature rises, the viscosity of the material decreases rapidly, and the gel does not form easily after extrusion. When the temperature drops, the viscosity increases rapidly, and it becomes necessary to apply a large extrusion force. Various problems may occur, such as plugging and extrusion of filaments with a curved [9] or an uneven diameter. To solve the problem of GelMA printability, methods employing two-step cross-linking, composite materials, GelMA microsphere formation [10], and microfluidics have been developed. For example, Wanjun Liu [11] used a coaxial nozzle to create a sodium alginate shell for a low-viscosity GelMA structure, allowing GelMA to undergo a second cross-linking with the support of cross-linked sodium alginate. Yong He [2] prepared a GelMA/nanoclay mixed ink that improved the printability of GelMA and obtained complex scaffolds with high precision. Although these methods have improved the printing capabilities of GelMA, they are ineffective when it is necessary to meet the new requirements of cell-laden printing. GelMA has a very low viscosity at 37 °C, which is cell-friendly. Using mixed materials to increase its viscosity will increase the extrusion force, resulting in low cell viability. The temperature and ionic cross-linking conditions required by the two-step cross-linking method will also directly affect cell survival. Therefore, it is important to find a suitable method to maintain the printability of GelMA while maintaining cell survival. A method to print low-viscosity bioink is key to solving this problem.

Embedded printing refers to a method of embedding a hydrogel into a second supporting hydrogel; it is also known as “Freeform Reversible Embedding of Suspended Hydrogels (FRESH) printing” [12], “Suspended Layer Additive Manufacturing (SLAM)” [13], etc. It was developed on the basis of extrusion 3D printing and was originally intended to break through the layer-by-layer deposition limitations of extrusion printing. The encapsulation of a bioink in the suspension bath allows the bioink to be printed at arbitrary and discrete positions so to form complex structures [14]. Besides, the suspension bath can mediate between the printability and the biological activity of the bioink and broaden the range of materials suitable for 3D bioprinting. Specifically, due to shear thinning properties and yield stress, the support bath can encapsulate very-low-viscosity bioinks preventing their spreading or gel collapsing and allowing sufficient time for crosslinking, so to stabilize the gel morphology and ensure cell viability. Commonly used suspension bath materials are gelatin, Carbopol, agarose, etc. Adam Feinberg a used mechanical force to pulverize gelatin into irregular particles as a support material and developed a technique called “FRESH” printing [12,15,16]. Bhattacharjee [17,18,19] mixed fluorescent polystyrene microspheres with PDMS to make printing materials and printed jellyfish, nesting dolls, and blood vessels using Carbopol as a suspension bath. Qunning Li [20] carried out a detailed study on the printing fidelity of GelMA in Carbopol. As a suspension bath, gelatin has temperature-sensitive properties. If the temperature of the bio-ink is high during the printing process, the stability of the support may be affected, resulting in a decrease in the viscosity around the sites of ink deposition. Carbopol requires changing the pH or adding a salt solution to liquefy the gel, both of which affect the activity of cells within the structure. As a suspension bath, agarose microgels not only have good biocompatibility, but also have a melting point of up to 95 °C, and their stability will not be affected by encapsulated high-temperature printing inks. The hydrogen bonds between agarose particles are sufficient to support a bioink, but the gel is very fragile macroscopically, can be destroyed by a small stress, and the printed structure can be released [13]. Therefore, agarose has very broad prospects for suspension printing.

In this study, a suspension bath of agarose particles was prepared to balance printability and cell viability, and a low-viscosity GelMA slurry was printed at a lower extrusion force to form 3D scaffolds. Senior [21] previously developed the SLAM technology using agarose particles and employed alginate to print structures such as spiders and hollow tubes. This research focused on the supporting effect of agarose on the formation of bio-ink and did not examined the suspension printing of GelMA, nor did it study the printing accuracy and effect of the material in an agarose suspension bath. Therefore, using the SLAM technology, a suspension bath of agarose particles was prepared in this study, and its rheological properties were studied. The filament diameter extrusion test of the GelMA slurry was carried out in the support bath, and a more suitable printing window was defined based on the GelMA/Agarose system; the shape and performance differences of the suspension printing structure and the extrusion printing structure were also compared. This study provides solutions for the low-viscosity printing of GelMA, presents the problems that need to be solved in embedding printing, and shows potential prospects in the field of embedded-printing customized scaffolds.

## 2. Materials and Methods

### 2.1. Materials Preparation

A small amount of LAP initiator (phenyl-2,4,6-trimethylbenzoyllithium phosphinate, EFL, Suzhou Intelligent Manufacturing Research Institute, Suzhou, China) was put into a brown bottle, a phosphate-buffered saline solution (PBS) was added, and the solution was heated in a 45 °C water bath for 15 min under shaking to prepare a 0.25% (*w*/*v*) LAP initiator standard solution. Gelatin–Methacryloyl (GelMA, EFL-GM-30, Suzhou Intelligent Manufacturing Research Institute, Suzhou, China) was placed in the LAP initiator standard solution, a small amount of red dye was added, and the solution was heated in a water bath at 60 °C for 1 h to fully dissolve the dye; 5.0%, 7.5%, 10% (*w*/*v*) GelMA solutions were prepared.

A certain amount of agarose powder was put into a beaker, deionized water was added, and the suspension was heated and stirred in a water bath at 98 °C. After dissolving the agar powder, the agarose solution was cooled into gels of different concentrations in the air. The cooled gels were put in a mixer (JYL-C50T, Joyoung Company, Hangzhou, China) and stirred for 120 s to obtain broken agar microgels. The broken microgels were poured into a centrifuge tube, centrifuged at 2000 r/min for 2 min to degas, and stored at 4 °C as suspension materials.

### 2.2. Rheological Characterization

Agarose microgels at different concentrations were tested for their rheological properties using a rheometer (Anton Paar MCR 102, Graz, Austria). The shear thinning properties were tested with linear rate sweeps over a shear rate range of 0.01–100 1/s. In the shear strain range of 0.001–100%, the frequency sweep test was performed according to the logarithmic variation law to obtain the storage modulus and loss modulus curves, and the yield stress value was obtained according to the curves. The thixotropy of the agarose gels was evaluated by increasing and decreasing shear on the agarose gels to obtain hysteresis loops at shear rates ranging from 0.01 to 100 1/s. All tests were carried out at room temperature (20 °C).

### 2.3. Fabrication Process of GelMA Scaffolds

#### 2.3.1. Process Parameter Test

The prepared GelMA solutions at different concentrations were put into 10 mL syringes, and the nozzles were installed. The syringes and the nozzles were shielded from light with adhesive tape and kept at 37 °C for use as extrusion materials. The prepared agarose gels of different concentrations were poured into a glass dish (diameter 60 mm) to a height of 7 mm (from the printing platform) and let cool to room temperature as suspension baths. The extrusion experiments focused on filaments’ diameter were carried out using a bioprinter (MAM-II, Fochif, Shanghai, China). During the experiment, a printer was used to control the process parameters, which are shown in Table 1. The height of the nozzle was set at 6 mm, a 35 mm filament was extruded in the X-axis direction, the filament diameter in the uniform part of the middle section was selected as the measurement object, a ruler was placed at the same height of the nozzle as a marker, and the camera was immediately placed at a fixed position after the extrusion was completed. Pictures were taken, processed with image-J software, and calibrated with a ruler, and the filament diameter was measured. The effect of different agarose concentrations, platform moving speed, extrusion speed, and nozzle size on the wire diameter was determined. The diameters of each group of filaments were measured five times and averaged.

Since the suspension bath can avoid GelMA collapse, layer heights of any size can be obtained in embedded printing. Considering that the following experiments involved the comparison of embedded printing and conventional printing, variables needed to be controlled in the structure of the stent; therefore, these experiments expand the research on layer height in conventional printing scaffolds in air and varies the parameters in the two processes at the same time. Printing of conventional scaffolds was performed at 20 °C. The side length of the bracket was 12 mm, the wire spacing was 1 mm, and 6 layers were printed.

#### 2.3.2. 3D Printing of Scaffold

The scaffolds were printed using GelMA at a concentration of 5%, and the parameters were obtained in Section 2.3.1. GelMA printed scaffolds were extruded in the substrate and agarose suspension bath and were named according to the process and filament spacing. For example, the scaffolds for suspension printing (600 μm, 800 μm, and 1000 μm) were called EP600, EP800, and EP1000, respectively, and the scaffolds for conventional extrusion (600 μm) were called AP600. During the printing process, a UV light source (365 nm) was set at a position 10 cm horizontally and at a certain height from the nozzle, and the light source was turned on for cross-linking when printing started. EP scaffolds were printed using GelMA incubated at 37 °C, and AP scaffolds were printed at 20 °C. After printing and curing, the EP scaffold was gently removed with a spatula, and deionized water was used to slowly rinse the residual agarose on the surface. After wiping with absorbent paper to absorb the surface moisture, the EP and AP scaffolds were placed in a −20 °C refrigerator to pre-freeze and shape for 1 h, and then into a freeze dryer to dry for 36 h.

### 2.4. Characterization of Scaffolds

After printing was completed, the size *d_p_* of the AP and EP scaffolds in the three directions of X, Y, and Z were measured by vernier calipers, and the deformation rate DR was obtained by comparison with the theoretical value *d*_0_ of the scaffold design. After freeze-drying, the scaffold was removed, and the size *d_c_* in the three directions of X, Y, and Z was measured again. The shrinkage rate SR was obtained by comparison with *d_p_*. This allowed judging the changes in the macrostructure of the stent during the printing process. DR and SR were determined by Equations (1) and (2), respectively. After the samples were sputter-coated with a gold foil for 300 s, the microstructure was observed using a field-emission scanning electron microscope (FESEM, JSM7600F, JEOL Ltd., Tokyo, Japan). The prepared scaffolds were subjected to Fourier transform infrared spectroscopy (FTIR) measurements (Bruker, Germany, spectral range 4000–450 cm/1; one step: 2 cm/s) to verify the scaffold composition.

(1) $$\mathrm{DR}=\frac{d_{p}-d_{0}}{d_{0}}\times 100\%$$

(2) $$\mathrm{SR}=\frac{d_{c}-d_{p}}{d_{p}}\times 100\%$$

### 2.5. Water Absorption Testing

This test was performed to measure the water storage capacity of the hydrogel scaffolds and determine the porosity inside the scaffolds. The scaffolds were cut into 4 pieces on average, and the dry weight (*W_d_*) of each sample was recorded. Then, the sample were soaked in deionized water, and every 10 min, after wiping off the surface water, their mass (*W_t_*) was recorded. The water absorption rate WA was determined by Equation (3)

(3) $$\mathrm{WA}=\frac{W_{t}-W_{d}}{W_{d}}\times 100\%$$

### 2.6. Mechanical Testing

The scaffolds were tested for their compression properties on an electronic dynamic and static fatigue testing machine (E1000, ITW Group Instron, High Wycombe, UK) at a speed of 1 mm/min. In addition to the mechanical testing of scaffolds with different structures (AP600, EP600, EP800 and EP1000), the effect of cross-linking conditions on the mechanical properties was explored using another experimental group. All stents were prepared with a side length of 12 mm and a thickness of 3.2 mm. A stress–strain curve was obtained by compressing the gels to 50% of their thickness, and the compressive modulus was determined from the slope of the linear region.

### 2.7. Statistical Analysis

The filament diameter extrusion experimental data are presented as mean ± standard error (SE, *n* = 5). Scaffolds fabricated from the same batch were used in characterization and performance testing to reduce errors, and experiments with scaffolds from different batches were not repeated (*n* = 1).

## 3. Results and Discussion

### 3.1. Rheological Testing

**Figure 1:**
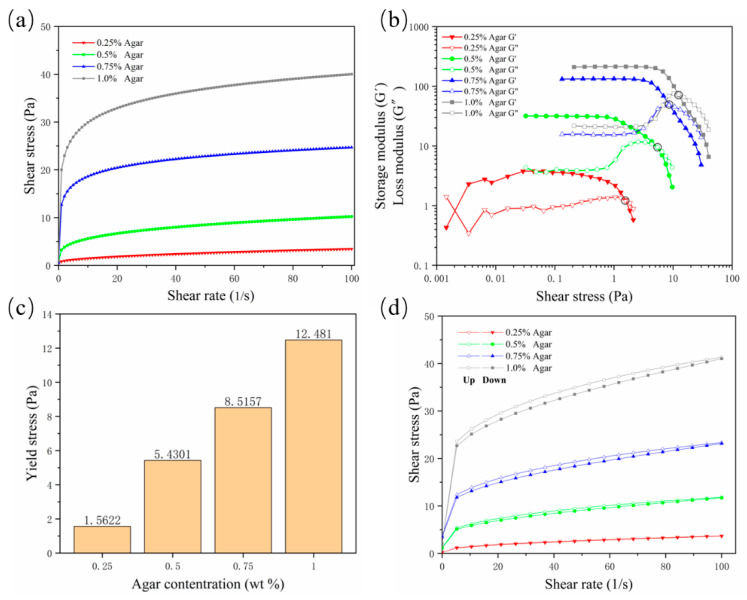
Rheological test results for four concentrations of agarose support bath. (**a**) Rate scan result, which intuitively shows the shear thinning characteristics; (**b**) storage modulus and loss modulus curves; (**c**) yield stress value; (**d**) thixotropy curve.

A suspension material needs to possess specific properties in order to suspend and encapsulate the printing material. The suspension medium should have the characteristics of a non-Newtonian fluid, similar to a solid when it is not stressed or is under low shear stress; as the shear stress increases, the viscosity of the suspension medium decreases, and the suspension medium begins to flow, becoming similar to a fluid; it needs to acquire a certain yield stress and shear-thinning properties. In this way, when the nozzle is embedded in the suspension medium and moves, the mechanical resistance of the nozzle is very small, and the hydrogel can be extruded; when the nozzle stops moving, the printed hydrogel can be fixed in the printing position by the suspension medium to complete the encapsulation. In this study, the shear-thinning properties of agarose gels were first validated. As the concentration increased, the viscosity of the gel increased continuously. The viscosity at a concentration of 0.25% was low, and the shear-thinning characteristics were not sufficient; they became sufficient from a concentration of 0.5% (Figure 1a, where “Agar” is the abbreviation of “Agarose”, the same below). The storage modulus (G′) and loss modulus (G″) curves well reflect the non-Newtonian fluid properties of the gel (Figure 1b). As the shear stress increased, the viscosity of the gel decreased, the storage modulus decreased, and the loss modulus increased. When G′ fell below G″, the gel began to flow, so the intersection of the modulus curves was used as the flow point to evaluate the yield value of different concentrations of agarose. The results showed that the yield strength at a concentration of 0.25% was too small, while starting from a concentration of 0.5%, the yield strength was detectable and increased with increased concentrations (Figure 1c).

In order to prevent the diffusion of printing materials and ensure good printing fidelity, the suspension medium should solidify rapidly to bridge the cracks or air pockets left after moving the nozzle. Thixotropy is the standard property evaluated to determine the speed of bridging. Because of the existence of thixotropy, the hydrogel used as support material can also be called “self-healing hydrogel”. The results showed that with the increase of the concentration, the area between the hysteresis curves of the agarose gel increased continuously, indicating that the gel’s rapid healing ability was decreasing (Figure 1d). In general, the yield value and thixotropy need to find a balance. Although a concentration of 0.25% is characterized by good thixotropy, the gel supporting ability is insufficient. Agarose at a concentration of 0.5% has suitable yield stress and thixotropy and is a potential optimal support bath formulation.

### 3.2. Process Test Result Analysis

**Figure 2:**
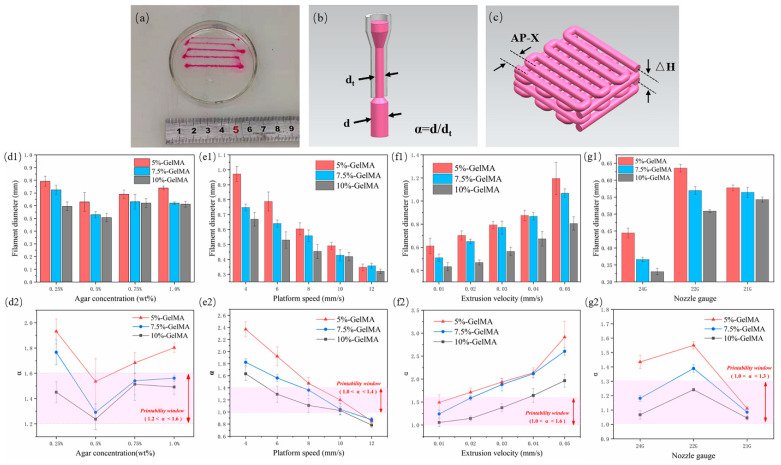
GelMA extrusion performance test results. (**a**) Actual graph of GelMA wire under different extrusion speeds; (**b**) schematic diagram of *α*; (**c**) schematic diagram of the scaffold layer height and wire spacing; (**d1**–**g1**) measurement results of the wire diameter for different agarose concentrations, platform moving speed, extrusion speed, and nozzle size, respectively, (**d2**–**g2**) corresponding *α* values and printability window.

The extruded filament diameter is an important factor determining the quality of the structure. In order to prepare scaffolds with good structure and morphology, we evaluated the extrusion performance of GelMA in regard to filament diameter (Figure 2a) and explored the variation of filament diameter under different process parameters, mainly agarose concentration, platform speed, extrusion velocity, and nozzle gauge. The fidelity is represented by α, that is the deviation between the actual wire diameter and the theoretical filament diameter (Figure 2b, *α* = *d*/*d_t_*, where *d* is the actual measured diameter, and *d_t_* is the inner diameter of the nozzle, which is the theoretical value).

First, three GelMAs were extruded at four concentrations of 0.25%, 0.5%, 0.75%, and 1.0% in an agarose suspension bath. For different concentrations of GelMA, 5% GelMA produced the largest filament diameter due to its low viscosity, which can produce greater expansion and deformation under the same extrusion force. Accordingly, 10% GelMA produced the smallest filament diameter. With the increase of agarose gel concentration, the filament diameter first decreased and then increased. It was the smallest at a concentration of 0.5% (Figure 2(d1)). At this concentration, the value of *α* was also the closest to 1, indicating the best fidelity. The reasons for the analysis are as follows: at 0.25% concentration, yield strength and viscosity were too low, and the solution was prone to diffusion and expansion. In addition to poor thixotropy, the support performance of 0.75% and 1.0% materials was lower than that at 0.5%, with the same gel stirring time, because at higher concentrations, the gel particles were more inhomogeneous. In this paper, 1.2 < *α* < 1.6 was selected as the printable window, and 0.5% agarose gel was found to be the optimal suspension bath concentration (Figure 2(d2)), which is consistent with the results of the rheological test.

Platform speed and extrusion speed are coupled parameters that affect the volume of deposited material. In this paper, the extrusion speed was first set to 0.02 mm/s, and the moving speed of the platform (nozzle) was changed. When the extrusion flow rate per unit time was constant, the larger the platform speed, the longer the extruded filament, and the smaller the filament diameter (Figure 2(e1)). *α* also conformed to this trend, and a value of 1.0 < *α* < 1.4 was selected as the printable window; 10 mm/s was the optimal platform moving speed (Figure 2(e2)). Then, we set the moving speed of the platform to 6 mm/s and change the extrusion velocity. When the extrusion flow rate per unit time increased and the wire length remained unchanged, the wire diameter continued to increase (Figure 2(f1)). We selected a value of 1.0 < *α* < 1.6 as the printable window and 0.01 mm/s as the optimal extrusion speed (Figure 2(f2)).

The nozzle has a basic role in extrusion printing. Extrusion experiments were also carried out with nozzles with different specifications. The inner diameters of 24 G, 22 G, and 21 G nozzles are 0.31 mm, 0.41 mm, and 0.52 mm, respectively. The denominator in the calculation of α changes accordingly. The results showed that for the 5% and 7.5% groups of GelMA, the 24 G nozzle extruded filaments with the smallest diameter, while the 22 G nozzle extruded slightly larger filaments than the 21 G (Figure 2(g1)). This may be due to the fact that when the extrusion flow per unit time was the same, the nozzle with a smaller diameter bore a greater shear force, and a greater deformation occurred when the nozzle was extruded, while the 24 G needle was too thin, resulting in a deformed filament diameter still smaller than those obtained with the 22 G and 21 G nozzles. The viscosity of 10% GelMA was higher, the degree of deformation after extrusion was lower, and the filament diameter with a 22 G nozzle was still smaller than that obtained with a 21 G nozzle. Therefore, in this paper, 1.0 < *α* < 1.3 was selected as the printable window (Figure 2(g2)), and the 21 G nozzle appeared to be the optimal extrusion nozzle gauge.

**Figure 3:**
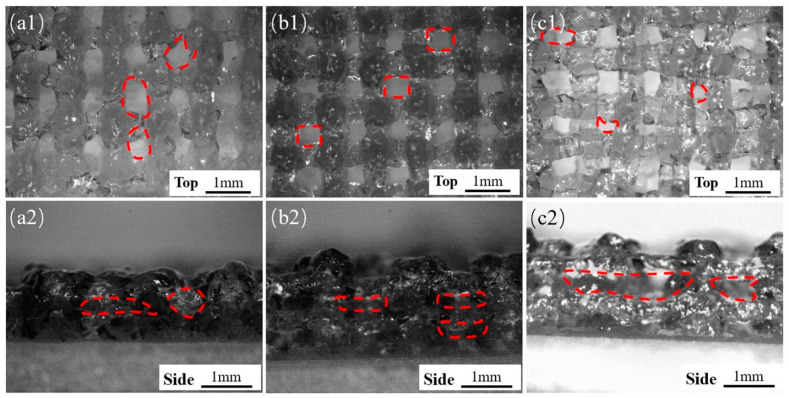
Shapes of the scaffolds with different layer heights under conventional extrusion printing (**a1**–**c1**, top views) for ΔH of 0.3 mm, 0.4 mm, and 0.5 mm, respectively, (**a2**–**c2**) side view.

In addition to the size of the extruded filament diameter, the layer height is also an important factor in determining the final structure (Figure 2c). Embedded printing can achieve any layer height. The front and side views of the extruded scaffold were observed with an optical microscope (B011, Supereyes, Shenzhen, China). With a story height ΔH = 0.3, hollows in the top view of the stent were obvious but uneven (Figure 3(a1)), and the side holes appeared with “big bottom and small top” (Figure 3(a2)). When the layer height was ΔH = 0.4, the top holes were clear and uniform (Figure 3(b1)), and the side holes were rectangular (Figure 3(b2)), indicating that ΔH was suitable. For ΔH = 0.5, the layer height was large, the filament could not be attached in time due to the excessive height, the nozzle was affected, and the top holes were very irregular. Therefore, we selected ΔH = 0.4 as the optimal layer height.

We finally selected these parameters: agarose concentration = 0.5%, platform speed = 10 mm/s, extrusion velocity = 0.01 mm/s, nozzle gauge = 21 G, layer height = 0.4 mm.

### 3.3. Structural Analysis of the Scaffolds

**Figure 4:**
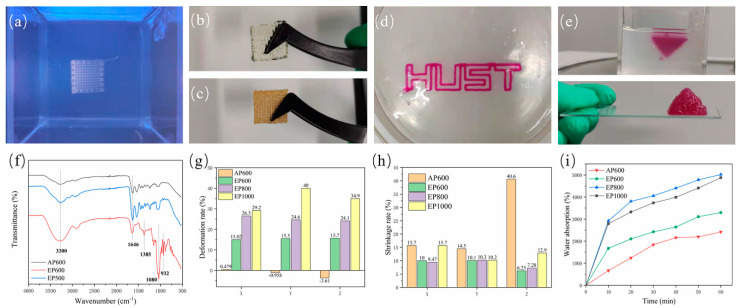
(**a**) Scaffold morphology in the support bath; (**b**) printed EP scaffold; (**c**) conventionally extruded scaffold; (**d**) extruded “HUST” hollow structure; (**e**) printed inverted triangle solid structure; (**f**) FTIR spectra of AP600, EP600, and EP500; (**g**) deformation rate after printing; (**h**) shrinkage rate after freeze-drying; (**i**) water absorption test for different scaffolds.

According to the parameters obtained in Section 3.2, scaffolds were prepared with different processes and specifications, such as AP600, EP600, EP800, EP1000. When the embedded printed scaffold was in the support bath, the structure was clear, and macroscopic voids were visible (Figure 4a). However, when taken out from the bath, due to the hydrophilicity of the hydrogel structure and the water contained in the suspension bath itself, agarose remained inside the stent during the molding process and was difficult to remove. Therefore, the EP scaffold did not clearly show a macroscopic porous structure (Figure 4b), while the conventionally extruded scaffold had a clear morphology after printing (Figure 4c); this may be a disadvantage of embedded printing. In theory, the formation of an internal reside can be avoided in two ways. First, by avoiding a fully clad structure so that the internal support bath residue can be cleaned directly (Figure 4d), but such structures are usually not strong enough and break when removed. Second, by reducing the filament spacing during printing, so that there is overlap between adjacent extruded filaments [22,23], that is, a pure solid structure is printed (as shown in the inverted pyramid in Figure 4e).

To this end, the EP500 scaffold was printed, and FTIR testing was performed using AP600 and EP600. The EP500 stent appeared as a solid stent with intersecting filaments, while the filaments of the AP600 and EP600 scaffolds were in contact with each other, and macroscopically appeared as a solid stent. The results showed that the structures of EP500 and AP600 (GelMA) were basically consistent, that is, the overlapping between filaments removed the internal agarose residues. However, the EP600 scaffold showed the characteristic peaks of GelMA and agarose at the same time, and the interference of agarose could not be completely ruled out (Figure 4f).

Embedded printing scaffolds will inevitably absorb moisture in the support bath and during the two steps of removing and cleaning the surface, and an agarose residue will remain inside them. The three factors can cause a great deformation of EP scaffolds. We considered the structural side length at the time of design plus the theoretical filament diameter × 1 as the theoretical side length, and the layer height × (layer number −1) plus theoretical filament diameter × 1 as the theoretical height to explore the deformation on the three directions of X, Y, and Z of the scaffold. The results showed that the side length base of the AP600 scaffold after printing was consistent with the designed structure, and the size in the Z-axis direction decreased a little due to the gravitational deposition of the material. However, the EP scaffolds showed large deformation after printing and removal. With the increase of filament spacing, the volume of the internal residual agarose also increased, the total water absorption was larger, and the deformation in all directions was also larger (Figure 4g).

Generally, scaffolds are stored by freeze-drying, and the freeze-drying process will cause shrinkage. The size of a scaffold after shrinkage is also a factor to be considered in tissue engineering. The results showed that the Z-direction shrinkage rate of AP scaffolds after freeze-drying was very large, and the scaffolds almost froze into flat squares. On the other hand, the EP scaffolds showed a lower overall shrinkage rate than the AP ones because of the internal residual agarose. Among them, EP1000 had the largest deformation due to water absorption during molding and also the largest shrinkage after freeze-drying (Figure 4h). In summary, reducing the filament spacing in suspension printing can reduce the interference of support materials and improve the printing structure.

It was observed experimentally that agarose can absorb water, which affects the overall structure of the scaffold. However, from another point of view, the presence of agarose can also improve the water absorption performance of the scaffold, thereby improving the metabolism of substances inside the scaffold. The water absorption rate of freeze-dried scaffolds was tested, and the quality of the scaffolds after water absorption at six nodes was measured for 1 h. Among them, AP600 showed the lowest water absorption. EP600 had the lowest water absorption among EP-type scaffolds, although it contained the highest number of GelMA fibers. EP800 showed the best water absorption performance, slightly better than that of EP1000 (Figure 4i). Because the agarose amount inside EP800 was similar to that in EP1000 and the number of GelMA fibers was higher than in EP1000, the comprehensive water absorption performance was higher.

**Figure 5:**
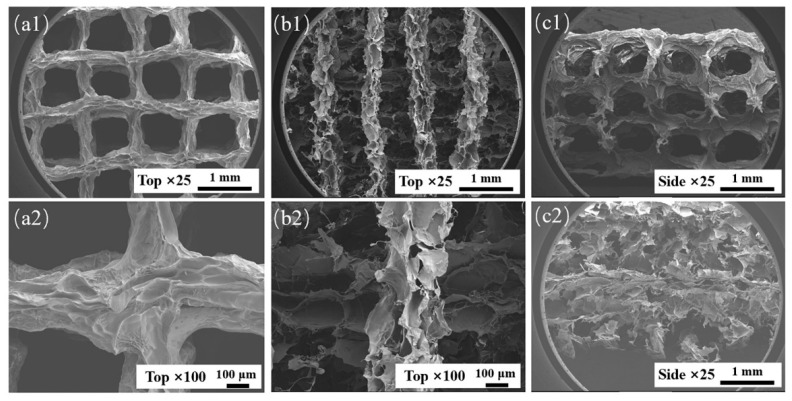
SEM images of AP1000 and EP1000, (**a1**,**a2**) top views of AP1000; (**b1**,**b2**) top views of EP1000; (**c1**) side views of AP1000; (**c2**) side views of EP1000.

During suspension printing, the surface of the extruded filament forms a solid–liquid (liquid–liquid) interface with the suspension bath, which is different from the gas–liquid interface during traditional extrusion. The surface of the extruded filament is subjected to different forces, which will affect its morphology. The microstructures of AP1000 and EP1000 scaffolds were observed. The results showed that the macroscopic pores of the AP1000 scaffold were clear and smooth, the microscopic surface was rough and presented continuous folds, and there were no pores reaching the interior (Figure 5(a1,a2,c1)). Several layers of filaments on the top of the EP1000 scaffold could be distinguished, and ere many other substances remained inside; this also confirmed the previous observation. The surface of the silk was a sheet-like discontinuous structure, with many holes and a large specific surface area (Figure 5(b1,b2,c2)). This structure may have an impact on strength but may be beneficial for cell adhesion growth and drug loading.

### 3.4. Mechanical Property Analysis

**Figure 6:**
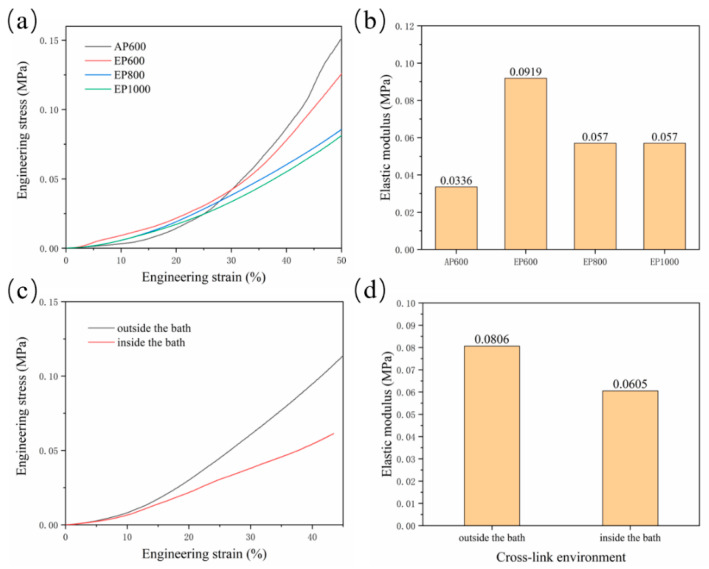
(**a**) Stress–strain curve of AP600, EP600, EP800, EP1000; (**b**) elastic modulus of the scaffolds; (**c**) stress–strain curve of EP600 in different cross-linking sites; (**d**) elasticity of the corresponding scaffold modulus.

Residual agarose inside the scaffold and the change of morphology of GelMA in the suspension bath affect the scaffold mechanical properties. The compressive properties of scaffolds with four configurations were tested. The results showed that the final stress of AP600 was the largest, indicating that the strength of the AP scaffold was higher (Figure 6a), which may be related to the structure of GelMA fibers. The EP scaffold had better elasticity, and with the increase of the number of GelMA fibers, the mechanical properties were also continuously enhanced; EP600 showed the highest elastic modulus (Figure 6b). On the whole, agarose can improve the elasticity of the scaffold inside the scaffold, but the overall strength is still determined by the GelMA fibers.

Another set of experiments were set up to explore the hindering effect of the support bath on light. A group of samples were removed and cross-linked in the suspension bath for 5 min after printing, while another group was kept in the suspension bath and cross-linked (for 5 min). The results showed that the strength and elastic modulus after cross-linking outside the suspension bath were higher (Figure 6c,d), indicating that the support bath has a certain influence on the illumination; thus, improving the transparency of the support bath is also important.

In summary, the suspension medium during the embedded printing process will have many effects on the scaffold, including on its surface morphology, water absorption properties, mechanical properties, etc. From another perspective, there are also positive effects. For example, changes in the apparent morphology of the scaffolds may improve drug loading and biocompatibility. The potential use of media inside the scaffolds to carry a second drug, cells, or as a reinforcing phase needs to be explored in future experiments.

## 4. Conclusions

In this study, GelMA 3D scaffolds were prepared by suspension printing using agarose particles as the support bath and GelMA as the printing ink. Through experiments, including rheological testing, process analysis, morphology analysis, and performance characterization, some issues about embedded printing scaffolds were explored. We found that 0.5% agarose microgel provided the best support performance. We also analyzed the printing suitability window of process parameters, the effect of the support bath on the microscopic topography of the scaffold surface and compared the mechanical properties of EP and AP. The overall strength of the embedded printed scaffolds decreased, but the presence of residual agarose could improve the elasticity and water absorption properties of the scaffolds. Therefore, this method explored some potential roles of the support bath in embedded printing and lays the groundwork for the preparation of good suspension structures, that can be applied in customized tissue engineering.

## Figures and Tables

**Table 1:** Values of the parameters in the extrusion experiments.

	Parameter	Agarose Concentration	Platform Speed	Extrusion Velocity	Nozzle Gauge
Group					
**Agarose concentration**		/	4 mm/s	0.02 mm/s	22 G
**Platform speed**		0.5%	/	0.02 mm/s	22 G
**Extrusion velocity**		0.5%	6 mm/s	/	22 G
**Nozzle gauge**		0.5%	6 mm/s	0.02 mm/s	/

## Data Availability

The data presented in this study are available on request from the corresponding author.

## References

[1] Bertsch P., Andrée L., Besheli N.H. (2022). Colloidal hydrogels made of gelatin nanoparticles exhibit fast stress relaxation at strains relevant for cell activity. Acta Biomater..

[2] Gao Q., Niu X., Shao L., Zhou L., Lin Z., Sun A., Fu J., Chen Z., Hu J., Liu Y. (2019). 3D printing of complex GelMA-based scaffolds with nanoclay. Biofabrication.

[3] Pan C., Han Y., Lu J. (2021). Structural Design of Vascular Stents: A Review. Micromachines.

[4] Shi L., Hu Y., Ullah M.W., Ullah I., Ou H., Zhang W., Xiong L., Zhang X. (2019). Cryogenic free-form extrusion bioprinting of decellularized small intestinal submucosa for potential applications in skin tissue engineering. Biofabrication.

[5] Shi L., Xiong L.M., Hu Y.Q., Li W.C., Chen Z.C., Liu K., Zhang X.L. (2018). Three-dimensional printing alginate/gelatin scaffolds as dermal substitutes for skin tissue engineering. Polym. Eng. Sci..

[6] Nocera A.D., Comin R., Salvatierra N.A., Cid M.P. (2018). Development of 3D printed fibrillar collagen scaffold for tissue engineering. Biomed. Microdevices.

[7] Van den Bulcke A.I., Bogdanov B., De Rooze N., Schacht E.H., Cornelissen M., Berghmans H. (2000). Structural and rheological properties of methacrylamide modified gelatin hydrogels. Biomacromolecules.

[8] Xie M., Yu K., Sun Y., Shao L., Nie J., Gao Q., Qiu J., Fu J., Chen Z., He Y. (2019). Protocols of 3D Bioprinting of Gelatin Methacryloyl Hydrogel Based Bioinks. J. Vis. Exp..

[9] Gao Q., He Y., Fu J.Z., Liu A., Ma L. (2015). Coaxial nozzle-assisted 3D bioprinting with built-in microchannels for nutrients delivery. Biomaterials.

[10] Chen H.N., Xing X.D., Tan H.P., Jia Y., Zhou T.L., Chen Y., Ling Z.H., Hu X.H. (2017). Covalently antibacterial alginate-chitosan hydrogel dressing integrated gelatin microspheres containing tetracycline hydrochloride for wound healing. Mater. Sci. Eng. C Mater. Biol. Appl..

[11] Liu W., Zhong Z., Hu N., Zhou Y., Maggio L., Miri A.K., Fragasso A., Jin X., Khademhosseini A., Zhang Y.S. (2018). Coaxial extrusion bioprinting of 3D microfibrous constructs with cell-favorable gelatin methacryloyl microenvironments. Biofabrication.

[12] Mirdamadi E., Tashman J.W., Shiwarski D.J., Palchesko R.N., Feinberg A.W. (2020). FRESH 3D Bioprinting a Full-Size Model of the Human Heart. ACS Biomater. Sci. Eng..

[13] Moxon S.R., Cooke M.E., Cox S.C., Snow M., Jeys L., Jones S.W., Smith A.M., Grover L.M. (2017). Suspended Manufacture of Biological Structures. Adv. Mater..

[14] McCormack A., Highley C.B., Leslie N.R., Melchels F.P.W. (2020). 3D Printing in Suspension Baths: Keeping the Promises of Bioprinting Afloat. Trends Biotechnol..

[15] Hinton T.J., Jallerat Q., Palchesko R.N., Park J.H., Grodzicki M.S., Shue H.J., Ramadan M.H., Hudson A.R., Feinberg A.W. (2015). Three-dimensional printing of complex biological structures by freeform reversible embedding of suspended hydrogels. Sci. Adv..

[16] Lee A., Hudson A.R., Shiwarski D.J., Tashman J.W., Hinton T.J., Yerneni S., Bliley J.M., Campbell P.G., Feinberg A.W. (2019). 3D bioprinting of collagen to rebuild components of the human heart. Science.

[17] Bhattacharjee T., Gil C.J., Marshall S.L., Uruena J.M., O’Bryan C.S., Carstens M., Keselowsky B., Palmer G.D., Ghivizzani S., Gibbs C.P. (2016). Liquid-like Solids Support Cells in 3D. ACS Biomater. Sci. Eng..

[18] Bhattacharjee T., Zehnder S.M., Rowe K.G., Jain S., Nixon R.M., Sawyer W.G., Angelini T.E. (2015). Writing in the granular gel medium. Sci. Adv..

[19] Hinton T.J., Hudson A., Pusch K., Lee A., Feinberg A.W. (2016). 3D Printing PDMS Elastomer in a Hydrophilic Support Bath via Freeform Reversible Embedding. ACS Biomater. Sci. Eng..

[20] Ning L., Mehta R., Cao C., Theus A., Tomov M., Zhu N., Weeks E.R., Bauser-Heaton H., Serpooshan V. (2020). Embedded 3D Bioprinting of Gelatin Methacryloyl-Based Constructs with Highly Tunable Structural Fidelity. ACS Appl. Mater. Interfaces.

[21] Senior J.J., Cooke M.E., Grover L.M., Smith A.M. (2019). Fabrication of Complex Hydrogel Structures Using Suspended Layer Additive Manufacturing (SLAM). Adv. Funct. Mater..

[22] Greenwood T.E., Hatch S.E., Colton M.B., Thomson S.L. (2021). 3D Printing Low-Stiffness Silicone within a Curable Support Matrix. Addit. Manuf..

[23] Romero R.G.T., Colton M.B., Thomson S.L. (2021). 3D-Printed Synthetic Vocal Fold Models. J. Voice.

